# Post-marketing risk analysis of bendamustine: a real-world approach based on the FAERS database

**DOI:** 10.3389/fphar.2024.1372401

**Published:** 2024-05-13

**Authors:** Dan Li, Yuan Zhang, Jia Qi Ni, Juan Zhu, Wen Ting Lu, Ya Lin Chen, Lei Cheng, Yu Qi Wang, Qian Jiang Li, Jie Wang, Yan Bing Lu, Jia Chen, Li Chen

**Affiliations:** ^1^ Zhejiang Provincial People’s Hospital Bijie Hospital, Bijie, Guizhou, China; ^2^ West China Second University Hospital, Sichuan University, Chengdu, Sichuan, China; ^3^ Key Laboratory of Birth Defects and Related Diseases of Women and Children, Ministry of Education, Sichuan University, Chengdu, Sichuan, China; ^4^ Department of Pharmacy, Evidence-Based Pharmacy Center, West China Second University Hospital, Sichuan University, Chengdu, Sichuan, China; ^5^ Department of Pharmacy, Chengdu Jinniu District People's Hospital, Chengdu, Sichuan, China; ^6^ Department of Pharmacology, Faculty of Medicine, University of Basque Country UPV/EHU, Leioa, Spain

**Keywords:** adverse events, data mining, post-marketing risk analysis, FAERS, pharmacovigilance, bendamustine

## Abstract

**Objective:** Bendamustine was approved for treating chronic lymphocytic leukemia and indolent B-cell non-Hodgkin lymphoma. Despite its therapeutic benefits, the long-term safety of bendamustine in a large population remains inadequately understood. This study evaluates the adverse events (AEs) associated with bendamustine, using a real-world pharmacovigilance database to support its clinical application.

**Methods:** We conducted a post-marketing risk analysis to assess the association between bendamustine and its AEs. Data were extracted from the US FDA’s Adverse Event Reporting System (FAERS), covering the period from January 2017 to September 2023. The characteristics of bendamustine-associated AEs and the onset time were further analyzed. Statistical analysis was performed using MYSQL 8.0, Navicat Premium 15, Microsoft EXCEL 2016, and Minitab 21.0.

**Results:** 9,461,874 reports were collected from the FAERS database, 9,131 identified bendamustine as the “primary suspected” drug. We identified 331 significant disproportionality preferred terms (PTs). Common AEs included pyrexia, neutropenia, infusion site reaction, progressive multifocal leukoencephalopathy (PML), injection site vasculitis, and pneumonia—all documented on bendamustine’s label. Notably, 16 unexpected and significant AEs were discovered, including hypogammaglobulinemia, which is concerning due to its potential to increase infection susceptibility following bendamustine treatment. Other significant findings were anaphylactic reactions, PML, and cutaneous malignancies, suggesting updates to the drug’s label may be necessary. Physicians should monitor for neurological and skin changes in patients and discontinue treatment if PML is suspected. Moreover, the median onset time for bendamustine-associated AEs was 13 days, with an interquartile range [IQR] of 0–59 days, predominantly occurring on the first day post-initiation. The β of bendamustine-related AEs suggested risk reduction over time.

**Conclusion:** Our study uncovered some potential pharmacovigilance signals for bendamustine, providing important insights for its safe and effective clinical use.

## 1 Introduction

Bendamustine is a bifunctional molecule combining alkylating and antimetabolic properties, which has demonstrated enhanced efficacy across various lymphoma pathologies. Numerous studies have documented the effectiveness of bendamustine, either alone or in combination with other agents, in treating conditions such as indolent non-Hodgkin lymphoma (NHL), chronic lymphocytic leukemia, and mantle cell lymphoma (Flinn, 2019; Wang et al., 2022). These treatments have been shown to improve progression-free survival, reduce residual tumor cells, and minimize tumor burden (Merryman et al., 2020).

In 2008, the U.S. Food and Drug Administration approved bendamustine for patients with rituximab-resistant indolent NHL and chronic lymphocytic leukemia (Lalic et al., 2022). Given its beneficial effects in treating relapsed or refractory hematologic malignancies and its synergistic potential with other antineoplastic agents, bendamustine has attracted significant recent interest due to its immunomodulatory effects (Palumbo et al., 2015; Arulogun et al., 2023; Gao et al., 2023). However, adverse effects such as fever, memory loss, anxiety, and rash have been noted during or after discontinuation of treatment (Yi et al., 2022). Furthermore, serious complications like progressive multifocal leukoencephalopathy (PML) have been reported, including a case in a patient with non-Hodgkin follicular lymphoma post hematopoietic stem cell transplantation and rituximab-bendamustine therapy, where JC Virus DNA was detected in both peripheral blood and cerebrospinal fluid (Uchida Trociukas et al., 2020). A retrospective study of 95 patients also highlighted non-prior chemotherapy as a significant risk factor for skin toxicities among NHL patients treated with bendamustine alone or in combination with rituximab (BR therapy).

Subsequently, on 24 March 2021, the UK Medicines and Health Products Regulatory Agency issued an advisory highlighting the increased risks of non-melanoma skin cancer and PML associated with bendamustine (GOV.UK, 2021).

Despite these insights, most safety data on bendamustine derive from clinical trials and literature reviews (Flinn et al., 2014; Shotton et al., 2024; Wang et al., 2023), with a lack of systematic research into AE signals based on extensive international and real-world databases. This study aims to fill that gap by analyzing AEs associated with bendamustine using the US FDA Adverse Event Reporting System (FAERS) database, providing valuable insights for its clinical application and future research.

## 2 Methods

### 2.1 Data source

We conducted an observational retrospective disproportionality analysis using a case/non-case study design to assess potential associations between bendamustine and various AEs (Almenoff et al., 2007; Bate and Evans, 2009). In this study, AEs associated with bendamustine were identified as signals when they were reported more frequently than other drug events in the background information within the database. We included all reports submitted to the FDA Adverse Event Reporting System (FAERS) from January 2017 to September 2023, reflecting the most recent update of the FAERS database at the time of our analysis.

### 2.2 Data filtering

Bendamustine, an antineoplastic drug approved by the FDA, was the primary suspect in the reports analyzed. We utilized fuzzy matching in MySQL on the “drug name” field to filter out reports specifically mentioning bendamustine and to remove duplicates. Key search terms included “Bendamustine,” “BELRAPZO,” “BENDEKA,” “TREANDA,” and “VIVIMUSTA.” AEs data within FAERS were coded according to the preferred terminology from the International Medical Dictionary for Regulatory Activities (MedDRA), version 26.1 (Zhang et al., 2023). MedDRA organizes terms into five hierarchical levels: System Organ Class (SOC), High-Level Group Terms (HLGT), High-Level Terms, Preferred Term (PT), and Lowe Level Terms (LLT), which facilitated the categorization and searchability of data at various levels (Shu et al., 2023).

### 2.3 Data analysis

Given the limitations of the FAERS database, specifically the absence of denominator data, we cannot directly calculate the incidence of AEs. However, disproportionality analysis serves as a robust method in pharmacovigilance to detect signals of disproportionate reporting related to bendamustine (Ahdi et al., 2023). In our study, we applied both Bayesian and Frequentist approaches to assess the association between bendamustine and AEs. We utilized several statistical measures, including the Reporting Odds Ratio (ROR), the Proportional Reporting Ratio (PRR), the Information Component (IC), and the Empirical Bayes Geometric Mean (EBGM) (Sakaeda et al., 2013; Moore et al., 2020; Yao et al., 2020).

A positive signal of disproportionality was defined according to several criteria: a PRR of at least two, a chi-squared value (χ^2^) of at least four, three or more reported cases, an IC05 > 0, EBGM>2 (Kinoshita et al., 2020). All statistical analyses were performed using Microsoft Excel 2016.

**FIGURE 1:**
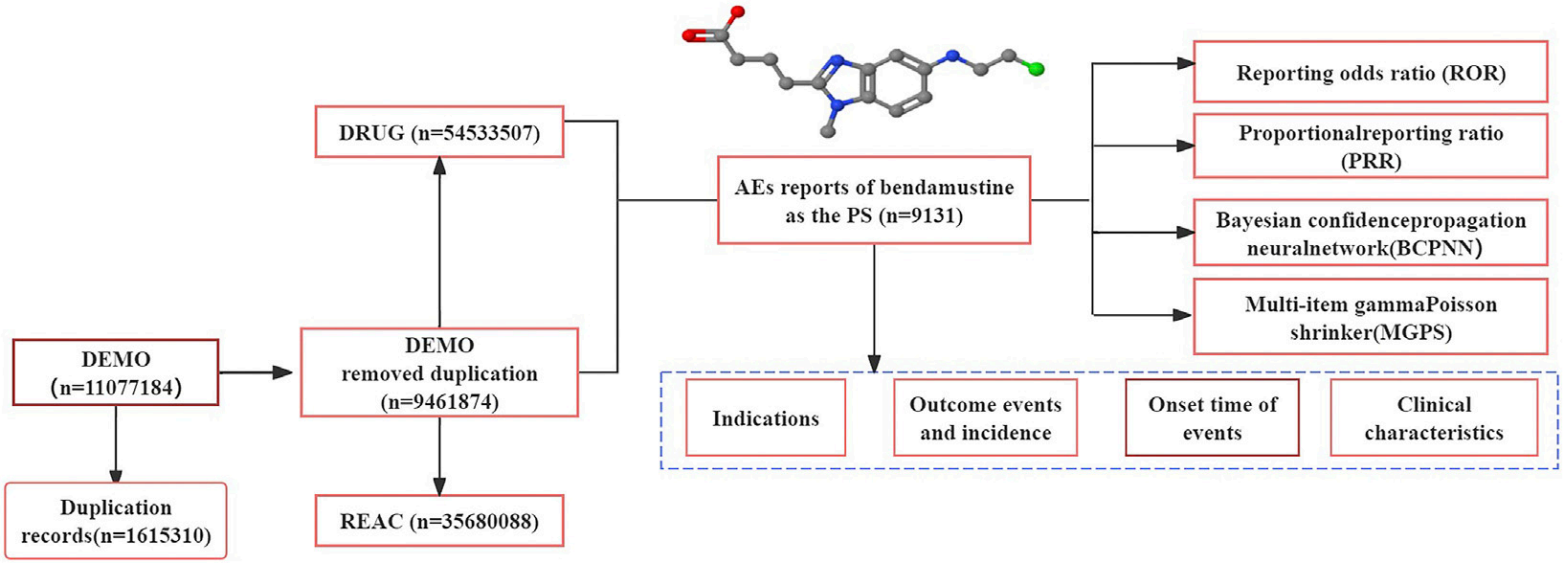
Process of retrieving bendamustine-associated AEs from the FAERS database. PS: primary suspected, AEs: adverse events.

Following the initial disproportionality analysis, detailed data concerning the AEs were collected, including patient characteristics (such as gender and age), reporting areas, indications for drug use, outcomes of the events, and the identity of the reporters. The methodology for data extraction, processing, and analysis is illustrated in Figure 1.

### 2.4 Time-to-onset analysis

To analyze the time-to-onset data, we employed median and quartile calculations along with Weibull shape parameter (WSP) tests (Chen et al., 2021). These analyses were conducted from the initial date of drug administration to the occurrence of specific AEs. We ensured the accuracy of our calculations by using only data that was fully reported with complete dates in the YYYYMMDD format, excluding cases with partial or missing dates (Zhao et al., 2023). Additionally, we removed instances with input errors, such as those where the AE occurrence was reported to precede the start of drug administration.

The tendency for AEs to occur was modeled using the two or three-parameter WSP. The tendency of AE occurrence can be predicted with the two or three-parameter WSP (Brucato et al., 2015; Mazhar Chiappini et al., 2021). The WSP is defined by two parameters: scale (α) and shape (β). A constant hazard over time corresponds to a β value of 1. Conversely, a β value less than one indicates a decreasing hazard over time, while a value greater than one suggests an increasing hazard. All analyses were performed using Minitab software (version 21.0; Minitab LLC, State College, PA, USA).

## 3 Results

### 3.1 Descriptive analysis

Our study analyzed 27 quarters of adverse drug event (ADE) data from the FAERS, spanning from the first quarter of January 2017 to September 2023. We extracted a total of 9,461,874 AEs reports from the FAERS database, and after removing duplicates, 9,131 reports were initially identified with bendamustine as the suspected drug. Further refinement by eliminating duplicate and misleading records reduced the sample to 5,195 reports for final analysis.

Demographic analysis revealed that patients over the age of 65 comprised 48.61% of the study sample, with a median age of 67 and an average age of 62.8 years. The data showed a higher incidence of AEs in male patients compared to females. Severe outcomes, including hospitalization and death, were reported in 2,406 cases, accounting for 30.38% and 20.41% of the severe reports, respectively.

The majority of AE reports were submitted by physicians, who contributed 45.44% of the data. This was followed by consumers (24.77%), other health professionals (13.85%), healthcare professionals (11.33%), and pharmacists (4.62%). The top five countries reporting the most bendamustine-associated AEs were the United States, Japan, France, Germany, and Britain, with Japan alone accounting for 33.49% of the cases. The summarized data and further details are presented in Table 1.

**TABLE 1:** Information of reports with bendamustine as the primary suspected drug from the FAERS database (January 2017 to September 2023).

	Bendamustine-associated AE reports (N = 9,131)		
Categories	Available number, n	Case number, n	Proportion of specific cases, %
Age (years old)	7,519	-	82.36
<18	-	298	3.96
18–64	-	2,782	37.00
≥65	-	4,439	59.04
Gender	8,230	-	90.13
Male	-	4,751	57.73
Female	-	3,479	42.27
Severe outcome	2,406		26.34
Death		491	20.41
Life-threatening		81	3.37
Hospitalization	-	731	30.38
Disability	-	15	0.62
Other severe outcomes	-	1,088	45.22
Reporters	8,968	-	98.21
Consumers	-	2,221	24.77
Healthcare professionals	-	1,016	11.33
Physicians	-	4,075	45.44
Pharmacists	-	414	4.62
Other health professionals	-	1,242	13.85
Reported countries (top 5)	5,957	-	65.23
US	-	1,906	32.00
Japan	-	1,995	33.49
Germany	-	798	13.40
France	-	677	11.36
Britain	-	581	9.75
Reporting year	9,130	-	99.99
2017	-	1,419	16.69
2018	-	1,524	17.10
2019	-	1,561	16.79
2020	-	1,533	10.20
2021	-	931	17.00
2022	-	1,552	6.68
Third quarter of 2023	-	610	0.01

### 3.2 Disproportionality analysis

**FIGURE 2:**
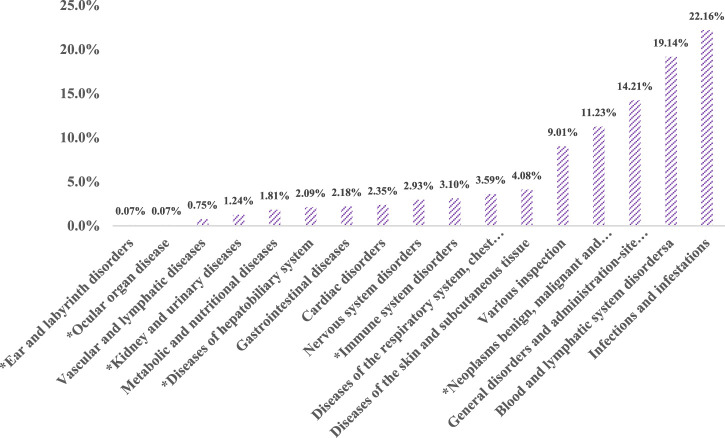
Proportion of bendamustine-associated AEs in different organ systems. Note: *New findings of bendamustine-associated AEs compared to the package insert of the drug.

In our examination of the FAERS database, signal strengths and bendamustine-related reports at the SOC level are detailed in Figure 2. We analysis identified statistically significant signals across 320 PTs within 17 SOCs, after excluding non-adverse drug reaction signals such as product issues, social environment factors, injuries, poisonings, operational complications, and effects from various surgical and medical procedures.

The SOC category “Infections and Infestations” reported the highest cumulative number of AE cases, accounting for 22.16% of all cases (945 cases). This was followed by disorders of the blood and lymphatic system (816 cases, 19.14%), and systemic diseases and reactions at the site of drug administration (606 cases, 14.21%).

A comparison with the AEs listed on the package insert for Cunda (bendamustine hydrochloride for injection, manufactured by Pharmachemie B.V.) revealed unexpected AEs affecting six organ systems. Notable among these were diseases of the blood and lymphatic system, which included six PTs such as embolism, hematoma, vasculitis, and vascular pain, accounting for a composition ratio of 0.75%. Ear and labyrinth disorders (PT: neurosensory deafness) and ocular diseases each accounted for 0.07% of reports.

Given their weak signals and relatively low report numbers, it is possible that false positives detected in these cases are due to the high sensitivity of the Reporting Odds Ratio (ROR) method (Gravel et al., 2023). Conversely, diseases of the liver and biliary system reported 89 cases (2.09%), with PTs such as acute hepatic failure, hepatic cirrhosis, abnormal hepatic function, hyperbilirubinemia, and veno-occlusive liver disease. The highest correlation and number of cases were seen in abnormal hepatic function (27 cases, χ^2^ = 117.32).

Furthermore, immune system diseases were identified in 132 cases (3.10%), encompassing 13 PTs including amyloidosis, anaphylactic reactions, cytokine release syndrome, cytokine storm, food allergy, graft versus host disease, hemophagocytic lymphohistiocytosis, and hypogammaglobulinemia. Diseases affecting the kidney and urinary system were reported in 53 cases (1.24%), including PTs like Waldenstrom’s macroglobulinemia and blastic plasmacytoid dendritic cell neoplasia.

### 3.3 The top 30 AE reports and signals

AE signals associated with bendamustine were thoroughly analyzed. To identify the top 30 AEs with high reporting rates and robust signals, effective signals were ranked in descending order based on the number of AE reports and the lower limit of the 95% Confidence Interval (CI) for the ROR, as shown in Table 2. Since the PRR values were equivalent to the ROR values, our signal strength analysis focused solely on comparing the ROR 95%CI lower limits (Chiappini et al., 2023).

**TABLE 2:** The Top 30 PT of BDM ADEs frequency and signal strength.

PT	Case(n)	95%CI lower limit	PT	Case(n)	*95%CI* lower limit
Pyrexia	186	4.03	Splenic marginal zone lymphoma recurrent *	3	430.06
Malignant neoplasm progression*	169	10.02	Waldenstrom’s macroglobulinemia recurrent *	4	345.13
Neutropenia	141	6.56	Infusion site phlebitis	9	316.96
Febrile neutropenia	118	11.91	Injection site vasculitis *	3	283.94
Lung infection	107	2.20	Coronavirus pneumonia	6	170.58
Thrombocytopenia	95	5.84	Injection site phlebitis	6	139.74
Disease progression*	88	5.02	Cytomegalovirus chorioretinitis	36	100.84
Anemia	75	2.72	Cytomegalovirus enterocolitis	14	88.12
Cytomegalovirus infection	66	23.05	Infusion site reaction	24	61.47
Neuropathy peripheral*	58	3.59	Leukemia cutis	3	60.83
Sepsis *	58	3.30	CD4 lymphocytes decreased	21	57.70
Neutrophil count decreased	57	8.37	Infusion site irritation	14	55.69
Pancytopenia	55	6.69	Paraneoplastic pemphigus *	3	52.23
Infection	53	2.16	Cytomegalovirus viremia	38	45.39
Platelet count decreased	52	2.89	Hypogammaglobulinemia	44	40.99
Lymphocyte count decreased	50	13.43	B-cell lymphoma recurrent	7	35.07
White blood cell count decreased	47	2.40	Progressive multifocal leukoencephalopathy	46	34.71
Progressive multifocal leukoencephalopathy	46	34.71	Aplasia pure red cell	17	34.01
Thrombocytopenia	44	17.72	Pneumonia cytomegaloviral	13	32.00
Hypogammaglobulinemia*	44	40.99	Autoimmune hemolytic anemia*	25	31.98
Septic shock*	44	6.27	Erythropoiesis abnormal	3	31.18
Chills	40	2.11	*Listeria* sepsis	3	29.01
Plasma cell myeloma *	40	4.81	Cytomegalovirus test positive	11	28.87
Blood lactate dehydrogenase increased *	39	18.75	Langerhans’ cell histiocytosis*	4	27.33
Cytomegalovirus viremia	38	45.39	Chronic lymphocytic leukemia recurrent	5	26.02
General physical health deterioration *	38	2.00	Diffuse large B-cell lymphoma refractory	7	25.55
Cytomegalovirus chorioretinitis *	36	100.84	Mantle cell lymphoma	9	24.97
Atrial fibrillation	32	1.858	Cytomegalovirus infection reactivation	21	24.50
Pneumocystis jirovecii pneumonia	32	14.72	Lymphoma transformation	3	23.82
Tumor lysis syndrome	32	18.96	Pneumonia *Escherichia*	3	23.32

Note: *New findings of bendamustine-associated AEs, compared to the package insert of the drug. PT: preferred term.

Comparison with the AEs listed in the current package insert for bendamustine revealed 16 new AEs. Among these newly identified AEs, the most significant were 169 cases of malignant neoplasm progression, 88 cases of disease progression, 58 cases of peripheral neuropathy, and 58 cases of sepsis. These findings are instrumental in providing a basis for potential updates to the AE listings in the bendamustine package insert.

### 3.4 Time-to-onset of bendamustine-associated AEs

**FIGURE 3:**
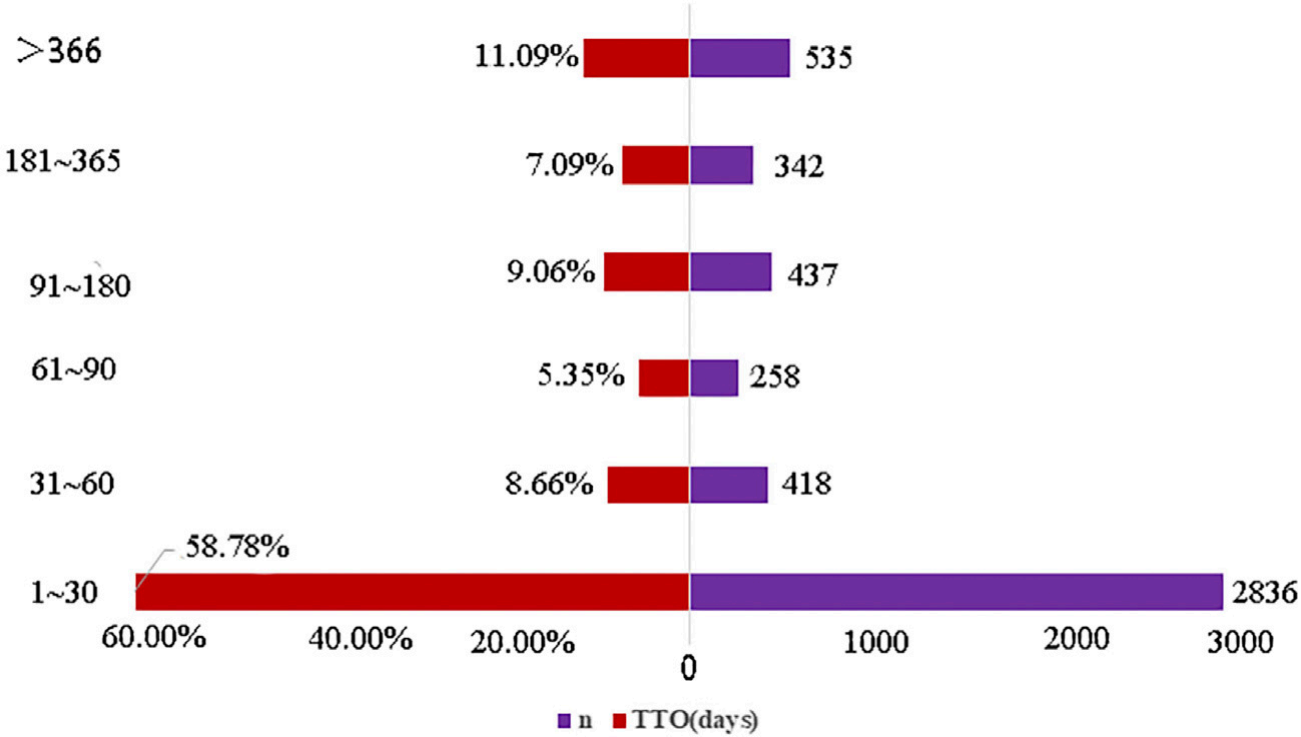
Time-to-onset of bendamustine-associated AEs.

**FIGURE 4:**
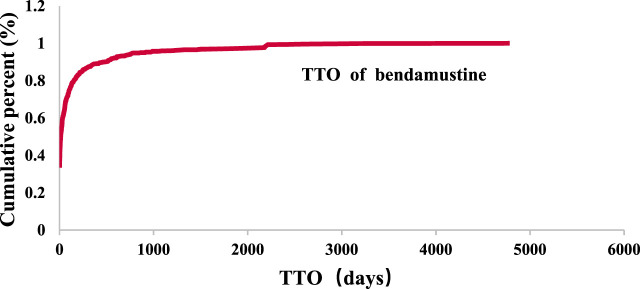
Cumulative distribution curve of TTO.

From the first quarter of 2017 to the third quarter of 2023, 4,825 cases reported at the SOC level included onset time data. The median onset time was identified as 13 days with an interquartile range (IQR) of 0–59 days, predominantly occurring on the first day post-initiation. The distribution of time-to-onset (TTO) for bendamustine-associated AEs was as follows: most occurred within the first month of treatment (n = 2,836, 58.78%), with diminishing frequencies in the second (n = 418, 8.66%) and third months (n = 258, 5.35%). Notably, approximately 11.09% of AEs (n = 535) were reported 1 year after initiating treatment with bendamustine (Figure 3). The cumulative proportion of these TTOs is depicted in Figure 4. Besides, as outlined in Table 3, the results revealed variable onset times for bendamustine-associated AEs across different SOCs. In the WSP analysis, all shape parameters β were less than 1, demonstrating that all AE signals in the SOC level had early failure types.

**TABLE 3:** Results of TTO analysis for signals at SOC level.

SOC	TTO(days)			Weibull distribution	
	Case(n)	Median (IQR)	Min-max	α (scale parameter)	β(Shape parameter)
Ear and labyrinth disorders	31	31(31–130)	0–731	2.80	0.74
Ocular organ disease	25	15(1–105)	0–1,268	3.12	0.73
Vascular and lymphatic diseases	93	7(0–113)	0–2,213	4.58	0.77
Kidney and urinary diseases	103	12(0–117)	0–4,784	3.80	0.74
Metabolic and nutritional diseases	131	12(0–117)	0–2,213	4.16	0.74
Diseases of hepatobiliary system	70	24.5(1–157.5)	0–2,160	3.32	0.73
Gastrointestinal diseases	321	13(0–79)	0–2,213	4.52	0.79
Cardiac disorders	123	20(1–147)	0–2,514	3.24	0.70
Nervous system disorders	177	31(0–211)	0–2,213	2.33	0.63
Immune system disorders	90	16(0–88.5)	0–2,213	5.02	0.80
Diseases of the respiratory system, chest and mediastinum	213	26(0–110)	0–2,514	4.11	0.77
Diseases of the skin and subcutaneous tissue	301	8(0–46)	0–2,183	5.54	0.82
Various inspection	399	10(0–101)	0–4,784	5.16	0.80
Neoplasms benign, malignant and unspecifed (including cysts and polyps)	276	46(0–437)	0–2,891	4.37	0.75
General disorders and administration-site conditions	621	9(0–72)	0–3,155	6.02	0.84
Blood and lymphatic system disordersa	456	19(0–90)	0–2,891	4.85	0.80
Infections and infestations	665	49(3–171)	0–3,155	4.09	0.76
^a^Various musculoskeletal and connective tissue diseases	76	14.5(0–245.5)	0–3,155	3.99	0.73
^a^Psychiatric disorders	45	133(7–242)	0–2,213	2.80	0.74

Note:

^a^ PTs, that are mentioned in the instructions but have no relevant valid signals after post-processing using four statistical methods. SOC: system organ class; IQR: interquartile range.

## 4 Discussion

Previous studies on bendamustine have predominantly focused on its mechanism of action, clinical trials, and literature reviews, with limited real-world research. Our study leverages the largest sample of real-world data to date, assessing post-marketing pharmacovigilance to identify new and significant AEs and evaluate the post-marketing safety of bendamustine for rational drug use.

The AEs associated with bendamustine were more commonly reported in males (57.73%) than in females (42.27%). A higher proportion of AEs occurred in elderly patients (n = 4,439, 59.04% of patients over 65 years old), likely due to the prevalence of B-cell lymphoma in middle-aged and older adults (Cesaretti et al., 2018). The clinical trial SEQUOIA (Tam et al., 2022) reported common hematological AEs such as decreased lymphocytes (87% incidence), neutrophils (83%), leukocytes (83%), and CD4 lymphocytes (77%), with Grade 3/4 decreases occurring in 77%–87% of patients. Nonhematologic AEs included nausea (73%), infusion-related reactions (63%), and constipation (50%), with severe AEs like febrile neutropenia and cytomegalovirus enterocolitis also reported, as described in the drug description of the package insert, and our study confirmed it.

Our analysis revealed that bendamustine induced AEs in 17 SOCs, including six systems not listed in the drug’s package insert. The most impacted was the immune system, followed by the kidney and urinary system. Notably, hypogammaglobulinemia presented the strongest signal (n = 44, χ^2^ = 2,274.55), with reports of persistent hypogammaglobulinemia in indolent NHL patients treated with bendamustine and rituximab (Sepulcri et al., 2021; Suzuki et al., 2022). Additionally, though nephrogenic diabetes insipidus had fewer reports (n = 4, χ^2^ = 117.48), it showed significant signal strength, with cases presenting symptoms like polyuria and low urinary osmolality shortly after treatment (Uwumugambi et al., 2016; Derman BA et al., 2017; Desjardins et al., 2022).

This comprehensive real-world analysis underscores the importance of monitoring for both expected and novel AEs in patients receiving bendamustine, enhancing our understanding of its safety profile and informing clinical practice.

It's worth noting that bendamustine most significantly prompted signals of infections, including bacterial and viral infections and viral activation. Due to the compromised immune function commonly observed in tumor patients, who often undergo radiation therapy, chemotherapy, and immunotherapy, it is challenging to distinctly attribute the high incidence of infections to therapy-induced lymphopenia or hypogammaglobulinemia. These infections may also stem from the underlying disease itself, suggesting a multifactorial etiology (Perriguey Sanchez-Gonzalez et al., 2021). Consequently, whether it's a bendamustine-induced ADR or the patient’s own underlying condition, physicians are advised to vigilantly monitor patients’ vital signs and immunoglobulin levels to preempt potential infections during bendamustine treatment is of the utmost importance.

Moreover, our study also found about severe signals of anaphylactic reaction induced by bendamustine(n = 22, χ^2^ = 36.10). Several reports have also highlighted bendamustine-induced anaphylactic reactions, presenting symptoms such as throat discomfort, pruritus, hives, general erythema, and facial swelling within 8 hours of administration (Sanchez-Gonzalez Bilò and Barbarroja-Escudero, 2014). Anaphylaxis represents a severe emergency medical condition that can escalate rapidly, potentially leading to death within minutes (Bilò et al., 2021). These reactions are not currently listed in the bendamustine’s package insert, posing a risk of being overlooked in clinical practice.

Conversely, while the package insert for bendamustine mentions psychiatric disorders and skeletal muscle-related AEs, our study did not detect active signals for these conditions. Only a few reports have linked bendamustine with AEs affecting these systems. Zimmer P et al. (Zimmer et al., 2015) observed that patients treated with bendamustine combined with rituximab reported a decline in cognitive perception, fatigue, and emotional functioning compared to those on conventional chemotherapy regimens (cyclophosphamide, doxorubicin, vincristine, prednisone). Additionally, research by Esposito et al. (Esposito et al., 2023) indicated a potential reduction in bone mineral density in patients treated with a chemotherapy regimen that includes bendamustine. However, these AEs could also be attributable to the concomitant use of rituximab, complicating the isolation of bendamustine’s specific effects. Given the inherent biases in self-reported FAERS data, clinicians must exercise caution when interpreting these findings and applying them to patient care. The identification of new and significant AEs not listed in the drug’s insert underscores the necessity of continual vigilance in the clinical monitoring of bendamustine use.

Recent findings have heightened concerns regarding bendamustine, particularly its association with non-melanoma skin cancers and progressive multifocal leukoencephalopathy (PML). In March 2021, the Medicines and Healthcare Products Regulatory Agency (MHRA) issued a risk warning following an increase in reported cases, highlighting the need for added precautions in the drug’s usage (MHRA, 2021). Our study validated these concerns, ranking PML 17th among the top 30 AE signals for bendamustine (showed as Table 2), with a significant number of reports (n = 6, χ^2^ = 1988.96, 95% CI lower limit = 34.71%). This supports the European review’s recommendation to include these risks in the Summary of Product Characteristics (SmPC), advising periodically monitor patients for skin changes in patients using a bendamustine-containing regimen (Rosas Cancio-Suarez et al., 2020).

A clinical retrospective study (Warsch et al., 2012) showed that during the period analyzed (7 January 2018, to 6 January 2020), 42 cases of PML were reported globally, including 11 deaths, compared to 9 cases in the previous period (7 January 2017 to 6 January 2018). Notably, 31 of these cases reported bendamustine as the most recent treatment prior to PML onset, underscoring a probable link to the drug, despite concurrent administration of other medications like rituximab and obituzumab. Given these findings, it is crucial for clinicians to remain vigilant for new or worsening neurological, cognitive, or behavioral signs that may suggest PML. If PML is suspected, bendamustine should be withheld until PML is ruled out. Diagnostic evaluations for PML should include Magnetic Resonance Imaging (MRI), lumbar puncture (cerebrospinal fluid John H. K. Cunningham Virus DNA test), and other tests. (https://www.medicines.org.uk/emc/product/9844/smpc).

Additionally, our analysis identified 95 cases related to skin system diseases from the standard MedDRA analysis query (SMQ), which included cutaneous malignant tumors like basal cell carcinoma (n = 17, χ^2^ = 101.90), squamous cell carcinoma (n = 6, χ^2^ = 33.73), and other serious skin reactions (shown as Table 4). Nonetheless, data from two clinical trials (the BRIGHT and GALLIUM trials) (Hiddemann et al., 2018; Flinn, 2019) have shown that the number of cases of non-melanoma skin cancers was higher in patients treated with a bendamustine-containing regimen compared to patients treated with other lymphoma regimens. Bendamustine causes prolonged lymphopenia and depletion of CD4-positive T cells. This effect was more significant when it was combined with rituximab. Our findings are consistent with clinical trial data suggesting an elevated risk of non-melanoma skin cancers in patients treated with bendamustine-containing regimens.

**TABLE 4:** SMQ classification of bendamustine-related skin diseases.

SMQ	PT	Cases	ROR	95%Cl low limits	X^2^
Malignant tumors of the skin	Basal cell carcinoma	17	7.92	4.91	101.90
	Lentigo maligna	3	59.33	18.93	168.93
	Neuroendocrine carcinoma of the skin	3	21.64	6.95	58.63
	Squamous cell carcinoma of the skin	6	7.5	3.36	33.73
Cutaneous premalignant diseases	Bowen’s disease	4	19.39	7.25	69.27
Serious adverse skin reactions	Keratoacanthoma	3	40.00	12.81	112.67
	Acute generalized exanthematous pustulosis	4	3.73	1.40	7.97
	Cutaneous vasculitis	3	7.63	2.46	17.24
	Dermatitis exfoliative generalized	8	13.11	6.54	88.90
	Drug reaction with eosinophilia and systemic symptoms	11	2.85	1.58	13.13
	Erythema multiforme	4	4.12	1.54	9.42
	Stevens-Johnson syndrome	18	9.24	5.81	131.08
	Toxic skin eruption	11	8.92	4.93	76.84

These results suggest a need for enhanced patient education and regular skin monitoring for those on bendamustine, with specific attention to suspicious skin changes. This approach should be integrated into the summary of product characteristics and patient information leaflets to mitigate the risk of severe AEs associated with bendamustine treatment.

Our study corroborated earlier findings (Czuczman et al., 2015) showing that most AEs associated with bendamustine occur early in the treatment course, typically within the first 3 months, with a median onset time of 13 days. The WSP test indicated an early failure type profile for all bendamustine-associated AEs at the SOC level, suggesting an increased risk of AEs shortly after treatment initiation, which then diminishes over time. Nonetheless, some AEs manifested after prolonged treatment, highlighting the need for extended follow-up in future clinical studies.

## 5 Limitations of the study

AE signal detection addresses the limitations of drug package inserts, such as delays in reporting, uncertainty, and incompleteness, by leveraging extensive data from spontaneous AE report databases to better reflect the drug’s safety profile (Feng et al., 2023). However, the reliance on the FAERS database, which primarily includes U.S. reports, introduces potential biases (Mai et al., 2015). Notably, the spontaneous nature of the reports often results in missing data, such as patient demographics, and the results may not be universally applicable due to population differences (Yin et al., 2022). Additionally, while methods like the ROR and PRR are straightforward, they are susceptible to false positives under certain conditions. Our analysis could not adjust for multiple confounding factors, such as concurrent drug use and patient comorbidities, which could influence AE outcomes.

Moreover, while the AE signals identified suggest an association between bendamustine use and potential AEs, they do not establish causality. This underscores the necessity for further post-marketing clinical trials to confirm these associations and to enhance the understanding of bendamustine’s safety profile.

## Data Availability

The original contributions presented in the study are included in the article/Supplementary Material, further inquiries can be directed to the corresponding authors.
